# Specifics of Pharmacokinetics and Biodistribution of 5-Fluorouracil Polymeric Complex

**DOI:** 10.3390/molecules28248096

**Published:** 2023-12-15

**Authors:** Olga V. Zhukova, Natalya A. Dubovskaya, Daria A. Zykova, Evgenia V. Arkhipova, Olga A. Vorobeva, Olga G. Zaborskaya, Sergey D. Zaitsev, Alexandra O. Grigoreva, Aleksandr A. Chicharov, Sergey A. Ryabov

**Affiliations:** 1 Department of Pharmaceutical Chemistry and Pharmacognosy, Federal State Budgetary Educational Institution of Higher Education, Privolzhsky Research Medical University of the Ministry of Health of the Russian Federation, 603950 Nizhny Novgorod, Russiazykova_d@pimunn.net (D.A.Z.); arhipova_e@pimunn.net (E.V.A.); vorobeva_o@pimunn.net (O.A.V.);; 2 Department of High-Molecular Compounds and Colloid Chemistry, National Research Lobachevsky State University, 603022 Nizhny Novgorod, Russiagrigoreva@chem.unn.ru (A.O.G.);

**Keywords:** polymers of methacrylic acid, 5-fluorouracil polymeric complex, pharmacokinetics of 5-fluorouracil polymeric complex, biodistribution of 5-fluorouracil polymeric complex

## Abstract

One of the promising and relevant directions in the treatment of oncological diseases is currently the development of a system for the delivery of antitumor drugs based on polyanions. Therefore, the aim of this work was to study the specifics of pharmacokinetics and biodistribution of a 5-Fluorouracil polymeric complex compared with commercial 5-Fluorouracil. Materials and methods: Monomeric methacrylic acid was used to synthesize polymers; 2-phenylpropane-2-ilbenzodithioate was used for the synthesis of poly(methacrylic acid). To study the molecular-weight characteristics of poly(methacrylic acid) by gel permeation chromatography, an experimental neoplasm model was obtained by grafting PC-1 cancer cells. Blood samples were drawn from the tail vein at different points in time. The rats were sacrificed via decapitation after drawing the last pharmacokinetic blood sample. To study the biodistribution, internal organs were isolated and analyzed. The measurements were carried out by high-performance liquid chromatography. Results: Our results demonstrate that incorporation in a polymeric complex changes the pharmacokinetics and biodistribution profile of 5-FU. The polymeric complex was shown to accumulate to a higher level in the lung and spleen. Conclusion: The results obtained are the basis for further studies to verify the efficacy of the 5-Fluorouracil polymeric complex.

## 1. Introduction

Chemotherapy is now widely used to treat cancer. However, current anticancer agents are distributed nonspecifically and inevitably affect biochemical processes in both cancer and normal cells. A huge number of side effects arise consequently, and negative therapeutic outcomes are not infrequent [1]. Increasing the selectivity of anticancer drugs is among the main problems of chemotherapy. The selectivity depends on whether the effective concentration of a drug accumulates at the site of its favorable effect and how long the effective concentration is maintained.

In view of the above, developing systems for the targeted delivery of anticancer drugs is the focus of numerous studies [2]. Researchers around the world are developing and studying various forms of polymer carriers of antitumor drugs. These are polymer nanogels capable of reversibly reacting to small external influences, liposomes [3], nanocrystals [4], nanoparticles [1,5,6,7], cocrystals [8] polymeric complexes [9], polymeric conjugates [10], micelles of amphiphilic polymers, etc.

To demonstrate the efficiency of an anticancer drug delivery system, it is necessary to study its pharmacokinetic characteristics and to verify its higher sensitivity to the site of disease (cancer tissues). 

5-Fluorouracil (5-FU) is a pyrimidine analog and provides one of the most effective anticancer agents, exerting a strong inhibitory effect towards a broad range of tumors [11,12]. 5-FU is most commonly used to treat neoplasms of the breast, gastrointestinal tract [13], liver, pancreas, and lung [14,15,16,17,18]. 

5-FU readily penetrates into the cell, sharing the facilitated transport system with uracil, and is rapidly converted to cytotoxic nucleotides [19]. To control cancer cell proliferation, 5-FU inhibits thymidylate synthase and the incorporation of its products in RNA and DNA, thus leading to cytotoxicity and cell death [20,21]. Pathological and normal cells are similarly entered and killed by 5-FU. 

Clinical use of 5-FU has been greatly limited by drug resistance in the past 50 years. For example, the overall response rate achieved with 5-FU alone is still only 10–15% in advanced colorectal cancer and merely increases to 40–50% when 5-FU is combined with other anticancer drugs. A search for new therapeutic strategies is therefore a pressing problem [16]. 

Various adverse effects are additionally caused by 5-FU, the set including dermatitis, mucositis, myelosuppression, and gastrointestinal disorders [1]. The adverse effects arise mostly because 5-FU lacks specificity towards cancer cells and has a short half-life in the plasma [22,23,24]. Various methods can be used to create delivery systems with 5-FU [25,26,27]. For example, by encapsulating 5-FU in a nanocarrier, it is possible to reduce or avoid the attack of dihydropyrimidine dehydrogenase on 5-FU. Thus, the effectiveness of 5-FU can be significantly improved, while the associated toxicity will be significantly reduced [19]. Also, some carriers have the ability to accumulate in the tumor focus and respond to local stimuli, such as a change in pH, leading to an accelerated release of the drug.

To reduce the 5-FU side effects and to improve the 5-FU therapeutic profile, 5-FU has been included in a polymer. The resulting polymeric complex was expected to improve the selectivity towards cancer tissues and thus increase the treatment efficacy and safety [28]. 

Various polymeric carriers to deliver anticancer agents are developed and investigated by many research teams worldwide [29,30]. 

Polymethacrylates are a widely available class of polymers. Polymethylmethacrylate (PMMA) is a cationic polymer, usually obtained by polymerization of methacrylic acid. The use of this polymer in the pharmaceutical industry can be divided into two categories. The first category includes its use in orthopedics, dentistry, plastics, ophthalmology, etc. The second category consists of different delivery systems: microparticles and nanoparticles [19,31,32], microspheres [33], and microgels [34] based on PMMA with controlled release [35].

Poly(methacrylic acid) (PMAA) and poly(acrylic acid) are water-soluble polymers and have carboxylic functional groups to allow their modification [28].

Poly(methacrylic acid) is a pH-sensitive polymer that is used for the manufacture of pH-sensitive carriers. So, in 2017, anionic–cationic polymethacrylate polymer mixtures and their effect on the release time of active substances from tablets were studied. Compared with conventional tablets, the authors demonstrated an increase in the release time of tablets with a polymer combination by more than 2 times [36].

PMAA microparticles with insulin for oral administration were studied. It was found that poly(methacrylic acid) exhibits a pH-dependent swelling due to the formation and dissociation of interpolymer complexes. Due to this, PMAA can be used for controlled release [37].

Also, the pH sensitivity of poly(methacrylic acid) is used to induce the destruction of polymer structures in acidic environments inside and/or outside tumor cells. In 2016, gold nanoparticles coated with poly(methacrylic acid) and combined with doxorubicin (DOX) as a model antitumor drug were developed. The experiments confirmed that the nanoparticles released DOX in a pH-sensitive manner and had an effective therapeutic effect [38].

Synthetic polyelectrolytes are known to act as adjuvants and to enhance the immune response when administered together with antigens, while lacking antigenic properties of their own. Immunostimulatory activity of polymeric adjuvants is based on their macromolecular nature. Immunostimulatory and, consequently, anticancer activities of polyanions are due to their direct effects and potentials to activate macrophages and to affect cytokine concentrations [39].

Previously, we studied the immunopharmacological effects of the methacrylic acid homopolymer on an in vivo tumor model. The concentrations of IL-6, IL-17 and TGF-β1 changed significantly and reached the level observed in intact rats. The concentration of IL-10 tended to normalize. The data obtained indicate that PMAA has a positive effect on the tumor process at an early stage of its growth [40].

PMAA with certain molecular-mass characteristics is promising as a means to construct an anticancer drug delivery system because PMAA intrinsically exerts the immunostimulatory effect and thus increases the anticancer effect of the cytostatic drug on cancer cells.

The objective of this work was to study the specifics of pharmacokinetics and biodistribution of a 5-FU polymeric complex compared with commercial 5-FU. 

## 2. Results

### 2.1. Characterization of 5-FU Polymeric Complex

**Scheme 1:**
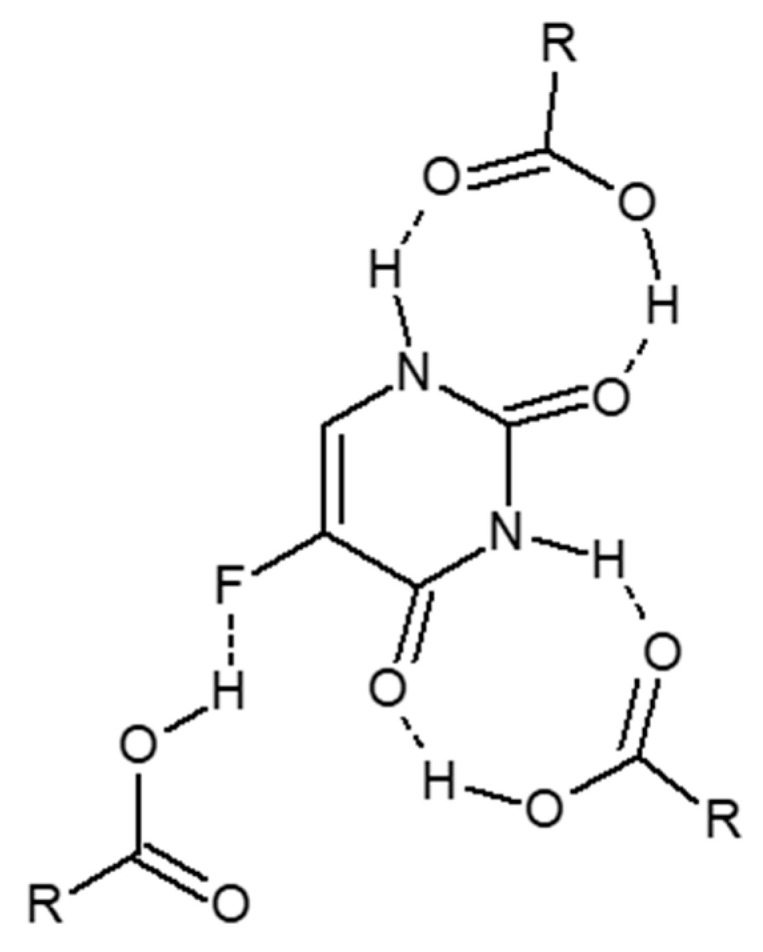
Intermolecular interactions of 5-FU with the carboxyl groups of PMAA. R, PMAA residue.

PMAA (Mn = 31.8 kDa, Ð = 1.17) was used as a carrier in the polymeric complex. Intermolecular interactions are determined by hydrogen bonding between the electronegative fluorine atom of 5-FU and the hydrogen atom of the PMAA carboxylic group that possesses a partial positive charge and is covalently bonded with an electronegative oxygen atom. Additional hydrogen bonds form between the hydrogen atom of the -NH group of the pyrimidine ring and an oxygen atom of a PMAA residue and between an oxygen atom and a hydrogen atom of the PMAA residue. This mechanism indicates that 5-FU is fully complementary to the PMAA residue. Each 5-FU molecule binds fully to two PMAA residues and, partly, a third PMAA residue (Scheme 1).

UV and IR spectroscopy, NMR confirmed the formation of an intermolecular complex of 5-FU with PMAA. 

**Figure 1:**
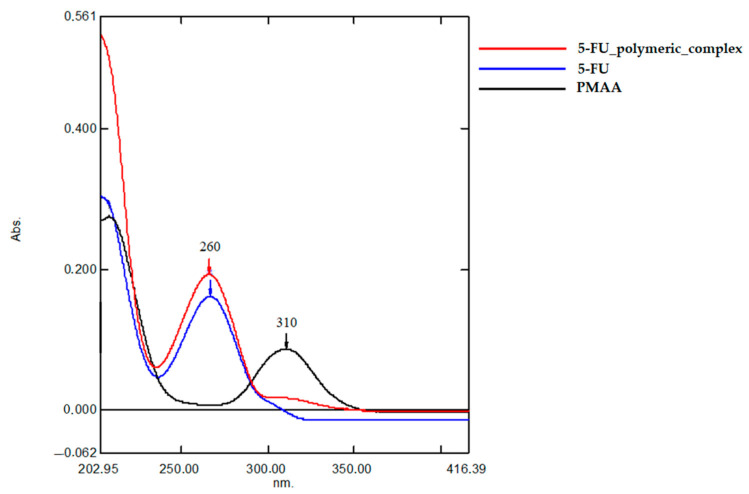
UV spectra of 5-FU (0.0001 g/mL (1 × 10^−4^ g/mL), a 100-fold dilution), PMAA (0.001 g/mL), and the PMAA complex with 5-FU (0.001 g/mL).

A peak at 260 nm is characteristic of 5-FU and is naturally absent from the spectrum of a polymer solution (Figure 1). High absorption at λ = 260 nm was observed in the UV spectrum of the polymeric complex, while absorption of a 5-FU solution was lower. A peak at 310 nm was characteristic of a polymer solution and was detected in the spectrum of the polymeric complex, demonstrating the presence of the polymer in the complex.

The degree of 5-FU incorporation in the polymeric complex was 92.01%.

**Figure 2:**
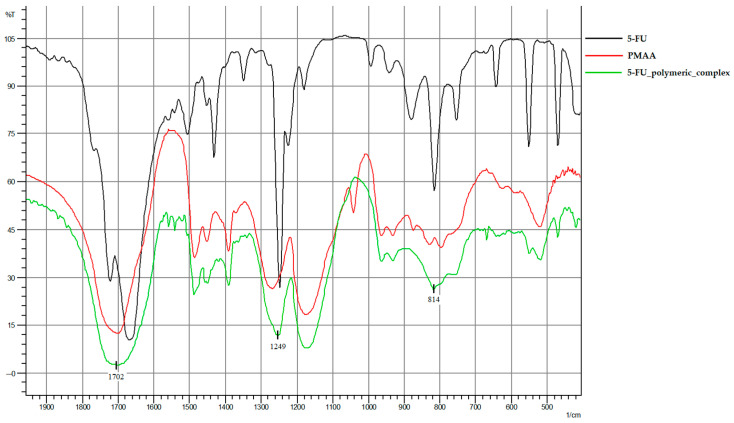
IR spectra of 5-FU, PMAA, and the polymeric complex.

IR absorption spectra of 5-FU, PMAA, and the polymeric complex are shown in Figure 2. Three peaks observed in the spectra in a range from 1702 to 814 cm^−1^ (Table 1) reflect the carbonyl group and two bond types, between fluorine and carbon atoms and between carbon and hydrogen atoms.

Evidence of the formation of the 5-FU polymeric complex is provided by NMR data. Shifts characteristic of the polymer and shifts characteristic of 5-FU are observed in the resulting 5-FU polymeric complex: 1H NMR 5-FU (DMSO-d6): 12.33 ppm (COOH), 11.51 ppm (NH), 10.73 ppm (NH), 7.75–7.74 ppm (CH).

### 2.2. Pharmacokinetic Studies

The pharmacokinetic distributions of the 5-FU polymeric complex and 5-FU were compared in a PC-1 tumor graft model in rats. Rats were injected intravenously with free 5-FU (4 mg/kg) or a polymeric complex (an equivalent 5-FU dose) once daily for 3 days. 5-FU is rapidly redistributed and rapidly eliminated after parenteral administration; its elimination half-life is only 8–20 min after intravenous administration [41]. Attempts are therefore made to design the 5-FU compositions that live longer in circulation [24]. 

When 5-FU was intravenously injected to rats, its concentration in the blood increased rapidly and substantially, and then shows an appreciably fast decrease, which corresponded to the start of 5-FU elimination from the body. Our findings agree with published data [42,43].

**Figure 3:**
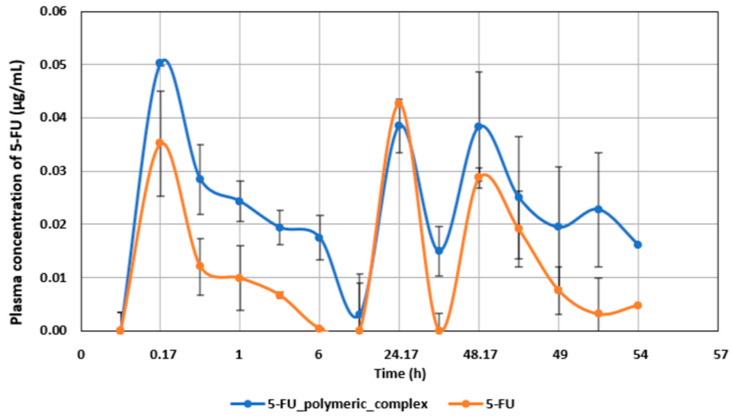
Pharmacokinetic profiles of 5-FU and the 5-FU polymeric complex in rats bearing PC-1 tumors. The results are shown as mean ± SD; each group included five rats.

Comparative pharmacokinetic profiling additionally showed that the retention time in plasma was higher in the case of the 5-FU polymeric complex. A thrice higher concentration was observed on day 3 of the experiment in the case of the polymeric complex. A maximum 5-FU concentration was achieved 10 min after administration with both polymeric complex and free 5-FU. By minute 360 (6 h), the 5-FU concentration decreased to 0.01616 mg/mL in the case of the polymeric complex and to 0.0048 mg/mL in the case of free 5-FU. The results were explained by the fact that PMAA was present in the 5-FU polymeric complex and substantially increased the molecular weight of the agent (Figure 3). Moreover, pharmacokinetic analysis showed that in the area under the curve (AUC) after the first injection, the 5-FU polymeric complex had more than 6 times more free 5-FU. 

Rats were injected intravenously with free 5-FU (4 mg/kg) or a 5-FU polymeric complex (an equivalent 5-FU dose) once daily for 3 days. The injection volume depended on the weight of the rat.

### 2.3. Biodistribution

Low-molecular-weight agents are rapidly eliminated from the body after intravenous administration and may consequently be nonspecifically distributed in normal tissues through capillaries. The biodistributions of the 5-FU polymeric complex and 5-FU were studied in vivo in a PC-1 tumor-bearing rat model. The therapeutic dose of 5-FU in humans is 12 mg/kg. The agent doses were calculated on a body weight basis, and the agents were injected in the tail vein in rats.

**Figure 4:**
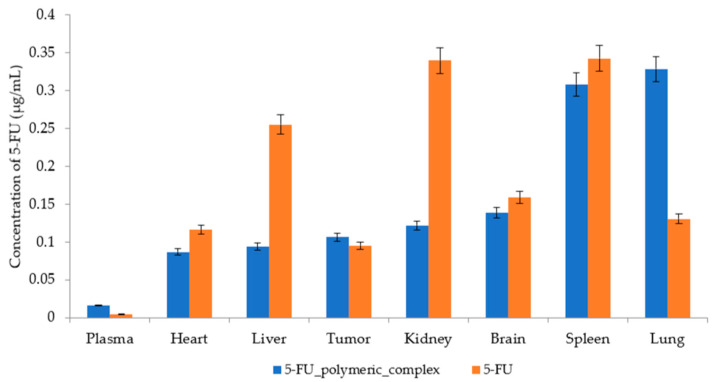
Tissue distributions of the 5-FU polymeric complex and 5-FU.

The 5-FU concentration was measured in the brain, liver, kidney, lung, tumor, heart, and spleen (Figure 4).

### 2.4. Comparison of the 5-FU Distribution Profile between Free 5-FU and the 5-FU Polymeric Complex

The PMAA-based 5-FU delivery system penetrates well into highly vascularized organs. Injected in circulation, the 5-FU polymeric complex is rapidly absorbed by plasma proteins and, in particular, opsonized by complement components [44]. Opsonized particles are recognized and captured by splenic and alveolar macrophages. Accumulation of the 5-FU polymeric complex was at its maximum in the spleen and lung, and minimum in the heart.

The concentrations of 5-FU and the 5-FU polymeric complex in the tumor were much the same on day 3 of the experiment (0.0956 and 0.1068 µg/mL, respectively). However, a thrice higher concentration in the blood plasma was observed in the case of the 5-FU polymeric complex than in the case of free 5-FU, suggesting a longer life in circulation and the possibility of delayed release for the 5-FU polymeric complex.

**Figure 5:**
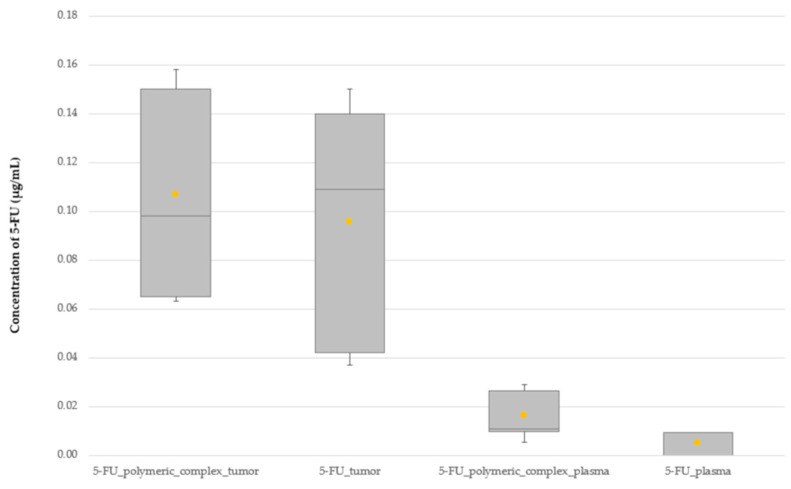
Distribution of free 5-FU and 5-FU polymeric complex in the plasma and tumor tissue. The differences between 5-FU polymeric complex and 5-FU in plasma were ns. The orange dots show the average values in the groups.

At the end of the experiment, the 5-FU concentration in the tumor was higher than in the plasma (Figure 5). The tumor-to-plasma concentration ratio reached 19.9, indicating that 5-FU can be rapidly redistributed from the plasma into the tumor.

The tumor-to-plasma concentration ratio of the 5-FU polymeric complex was 6.59 (Figure 5), indicating that it took far longer for the complex to be redistributed from the plasma into the tumor. Moreover, the plasma concentration of the complex was 3.4 times higher than that of free 5-FU, and the drug might have further acted longer.

The results indicate that the 5-FU polymeric complex must remain in the tumor longer than free 5-FU, displaying a delayed release profile. 

**Figure 6:**
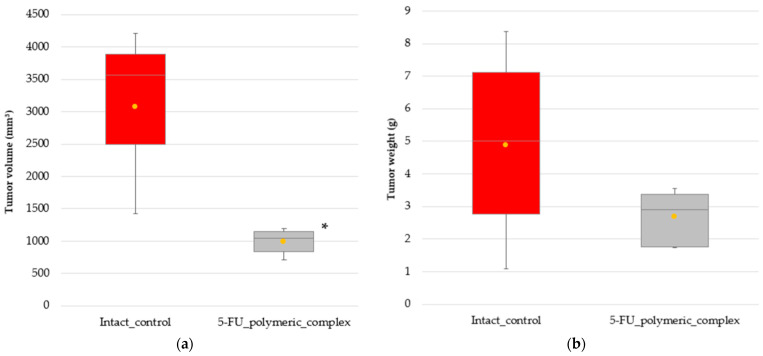
(**a**) Tumor volume, mm^3^; (**b**) tumor weight, g. Differences in tumor volume between intact rats and rats treated with the 5-FU polymeric complex (*) were significant at *p* < 0.05. Rats were injected intravenously with the 5-FU polymeric complex (an equivalent 5-FU dose—4 mg/kg) once daily for 3 days. The injection volume depended on the weight of the rat. The orange dots show the average values in the groups.

The tumor volume and weight in rats treated with the 5-FU polymeric complex were substantially lower than in untreated rats (intact control) (Figure 6a,b).

The average values of the volume and weight of tumors of intact laboratory animals and laboratory animals injected with the 5-FU polymeric complex are shown in Table 2.

## 3. Discussion

As a result of the high metabolic rate of the drug in the liver tissues, as well as in the blood, where 5-FU is converted into dihydrofluorouracil (under the action of the enzyme dihydropyrimidine dehydrogenase), the question is raised about ways to maintain therapeutic plasma concentrations without constant administration of the drug in high doses, which can cause serious side effects when the critical concentration limit of 5-FU is exceeded. Therefore, one of the most important goals of pharmaceutical research is the development of delivery systems of 5-FU to reduce toxicity and increase the specificity of the resulting system [19]. 

In many studies where 5-fluorouracil delivery systems are being created, the authors note a change in the distribution of delivery systems in contrast to free 5-FU [43,45,46].

This occurs as a result of changes in the physical and chemical characteristics of 5-FU due to the addition of a carrier, which, consequently, changes the pharmacokinetics and biodistribution.

According to the obtained pharmacokinetic data, with simultaneous administration of the 5-FU polymeric complex and free 5-FU to groups of laboratory animals, the concentration of free 5-FU decreased rapidly. By 6 h after the injection, it was practically undetectable (0.0004 µg/mL). These data correlate with another study in which a polymer prodrug of 5-FU was obtained and then tested in vivo in comparison with free 5-FU. After intravenous administration, 5-FU is rapidly removed from the circulation, which led to complete removal 6~8 h after administration [43].

In our study, the concentration of the 5-FU polymeric complex remained at a relatively high level by 6 h after injection (0.0175 µg/mL). Thus, 6 h after injection, the concentration of the 5-FU polymeric complex was more than 43 times higher than the concentration of free 5-FU.

Polymeric systems with antitumor drugs in their composition increase the selectivity and specificity of the action of drugs to tumor cells. Currently, along with chemotherapy, immunotherapy is of particular importance in the treatment of tumor diseases.

Polymeric particles are of interest in this direction, given the fact that they are used as carriers for antitumor agents. In the course of this study, an assessment of a polymeric complex based on low-molecular-weight PMAA was carried out, for which an immunopharmacological effect is noted [30,40,47,48,49].

And since the increased content of 5-FU in the 5-FU polymeric complex in the spleen and lungs may indicate the accumulation of this complex in organs precisely because of PMAA, and not because of 5-FU, this may be a positive factor in the activation of macrophages, which requires further clarification.

The spleen is a blood depot in which T- and B-lymphocytes accumulate, and erythrocytes are destroyed and absorbed by macrophages. Due to its anatomical structure and good blood supply, the spleen can participate in the metabolism of drugs with a large molecular weight and/or a pronounced charge, which is confirmed during the experiment. Being in the blood, the 5-FU polymeric complex, when passing through the spleen, could be captured by macrophages of the organ, as well as bind to B-lymphocytes, which leads to the accumulation of the complex in higher amounts in the spleen, which is not observed in low-molecular-weight 5-fluorouracil [50].

We also obtained an increase in the accumulation of 5-FU in the form of a polymer complex in lung tissues compared to 5-FU by more than 2 times. The results of another study fully agree with these data, where the distribution of gelatin microspheres loaded with 5-FU was studied by organ. Gelatin microspheres loaded with 5-FU efficiently delivered 5-FU to the lungs compared to free 5-FU. This was confirmed by the percentage of 5-FU distributed to the lungs using gelatin microspheres, which was 2 times higher than after the use of free 5-FU [51].

This can be explained by the fact that polymer-based 5-FU delivery systems penetrate well into highly vascularized organs. Once in the bloodstream, due to the large molecular weight and negative charge of the polymeric complex, polymer delivery systems quickly adsorb plasma proteins, including opsonized components of the complement system. Cells of various organs and tissues of the monocyte–macrophage system are able to recognize and phagocyte opsonized elements. Although the liver is considered the most important organ in the process of phagocytosis, it also involves the spleen, lungs, etc. As a result, the intracellular concentration after the introduction of 5-FU in the polymer-based delivery system is several times higher than the concentration achieved with the introduction of low-molecular-weight 5-FU [52].

The data obtained suggest that polymer-based 5-FU delivery systems may be suitable for the treatment of lung cancer, which requires further study and refinement.

There was also an increase in the content of 5-FU in the polymeric complex in the tumor by 1.2 times compared to pure 5-FU. These results of increased localization of 5-FU in the composition of complexes in tumor tissue are consistent with the results of other studies. For example, Wang W. et al., studying liposomal polymeric complexes based on 5-fluorouracil, noted a statistically greater accumulation of liposomal 5-FU in the tumor [53].

When studying the biodistribution of N-succinyl chitosan nanoparticles loaded with 5-fluorouracil, the researchers also revealed an increased accumulation of 5-FU nanoparticles in the tumor compared with free 5-FU [54].

This circumstance can be explained by the “loose” structure of the tumor tissue, as well as the leaky vascular network. Compared to typical well-organized arteries, tumor angiogenesis contributed to drug retention due to high vascular density and permeability, defective vascular architecture and poor lymph outflow from interstitial spaces of tumor tissue. All this allows high-molecular-weight substances to accumulate in the tumor tissue [55,56,57,58].

That is why the development of systems for the delivery of antitumor drugs is currently a promising and relevant direction in the treatment of oncological diseases.

In our work, the object of research was a polymeric complex based on PMAA.

Poly(methacrylic acid) changes the pharmacokinetics of the drug, and increases the circulation time of the complex in the circulatory system, which is consistent with the previously obtained data. The tumor growth rate was evaluated during therapy with branched polymer systems based on poly(methacrylic acid) and cellulose. The researchers demonstrated fluorescent imaging, which showed the ability of prepared branched polymer systems to provide a permeability and retention effect [59].

This can be explained by the change in the molecular weight of the polymer system in comparison with the free low-molecular-weight 5-FU and the loose structure of the tumor tissue, in which the vascular system is intermittent. Vascularized tumors, as well as some vascularized metastatic tumor nodules, have an increased permeability and retention effect. These properties of tumor tissues can be used to develop “passive” delivery systems.

On the other hand, the increased selectivity of polymethacrylic delivery systems can also be explained by the fact that poly(methacrylic acid) is a pH-sensitive polymer nanocarrier. The altered pH value observed in pathological conditions, including cancer or inflammation, is widely used to trigger the release of drug molecules into the desired biological organ (e.g., gastrointestinal tract) or intracellular compartment (e.g., lysosome or endosome). It is known that the pH value in tumor tissues is acidic by nature (pH 6.5), almost one full pH unit lower than the pH of blood (pH 7.4). In addition, a decrease in pH is observed in intracellular compartments, such as endosomes and lysosomes with pH values of 5.5–5.0, respectively. When poly(methacrylic acid) is used as part of the delivery system at an acidic pH value, the drug is released by reducing the electrostatic interactions of the cationic drug and the anionic polymer due to protonation of polymer carboxylate groups [60].

This is confirmed by a number of studies, for example, in one study, the ability of a new system of nanoparticles based on starch terpolymer, poly(methacrylic acid) and polysorbate 80 to load and release doxorubicin (Dox) depending on pH was investigated. It was found that anionic particles are able to efficiently load large amounts of cationic Dox, while the rate of drug release depended on the pH value. The rate was significantly higher at a slightly acidic pH than at a neutral one, which ensures a different release of the drug between the tumor tissue and normal tissue. A delivery system that significantly accelerates the release of the drug in an acidic environment can help reduce the toxicity of the drug to normal tissues while increasing the chemotherapeutic effect on the tumor [61].

In another study, hydrogels with methacrylic acid were studied for the enhanced targeted delivery of 5-fluorouracil to colon cancer cells. The data confirm that such a drug delivery system reacts to the acidity inside the tumor cells and releases drugs in a regulated manner [55,62].

In addition, it is interesting to note that not only PMAA has a pH-dependent release of the drug. Polymethylmethacrylate is also widely used by researchers in the development of delivery systems for the controlled release of anticancer medicaments [32,33,34]. For example, in a study of loaded 5-FU β-cyclodextrin-containing polymer nanoparticles of samarium ferrite coated with polymethylmethacrylate, researchers demonstrated increased release of the drug from nanoparticles with a decrease in pH to 6.0 and below [32].

In another study, an oral form of 5-FU delivery for the colon was obtained using the method of imprinting the molecular surface with chitosan–polymethylmethacrylate as matrix microspheres and with 5-FU as a matrix molecule. As a result of modeling the gastrointestinal tract system, the authors found that the mechanism of release in vitro strongly depended on pH and time. 5-FU was not released at pH 1.2, slow release was observed at pH 6.8, and rapid release was achieved in the simulated colon fluid. This can be explained by the fact that the degree of protonation of the 5-FU amine decreased with increasing pH, so that the electrostatic force between the microspheres, imprinted cavities, and the 5-FU matrix molecule weakened, which led to an increase in the release rate of the drug [33]

The use of acidic pH as a trigger of the tumor microenvironment has certain disadvantages. This, in turn, has led to the emergence of other delivery systems, such as nanoparticles and liposomes, showing good results. The emergence of drug delivery systems based on nanoparticles and liposomes has contributed to improving the delivery of low-molecular-weight drugs, which is confirmed in studies of recent decades. For example, in 2009, N-succinyl-chitosan (5-FU-Suc-Chi/NP) nanoparticles loaded with 5-fluorouracil were studied. The pharmacokinetic data obtained during this study showed that nanoparticles could circulate in the circulatory system for 4 days, while the amount of 5-FU-Suc-Chi/NP at the tumor site increased as the circulation time increased. Thus, the 5-FU included in the nanoparticles demonstrated delayed release, implying a long systemic delay in the circulatory system. This is also confirmed by the biodistribution data, which indicated an increased directivity of nanoparticles compared to low-molecular-weight 5-FU to the tumor [54].

The use of delivery systems makes it possible to improve a number of biopharmaceutical characteristics of antitumor drugs, allowing on the one hand to increase the effectiveness of the drug, and on the other hand to reduce the toxic effect on the body.

This conclusion is consistent with the data of another experiment where Dox was used as an antitumor drug, which was loaded into heparin-based nanoparticles. Loaded cross-linked nanoparticles had a longer circulation time in the circulatory system, and had excellent accumulation in the tumor compared to free Dox [63].

Various cisplatin nanoparticles based on various polyglutamic acid copolymers (polymer micelles) have also been proposed. Pharmacokinetics and biodistribution measurements have shown that cisplatin nanoparticles with polyglutamic acid and polyethylene glycol copolymer are characterized by a long circulation time in the blood, as well as selective and significant accumulation in Lewis tumor and lung carcinoma. The platinum concentration in mice with lung carcinoma treated with these nanoparticles remained 46 times higher than in mice treated with equivalent doses of free cisplatin [64,65].

The advantage of our polymer system, represented by a true solution, is the simplicity of its preparation, compared with nanoparticles and liposomes. At the same time, the data obtained in the course of our study correlate with the data obtained in the course of studies of other systems of delivery of antitumor drugs. Based on the results of the experiment, we received the following tasks to solve and explain:

1. For what reason is there accumulation of the delivery system in the lungs, and is it possible to use this accumulation for the treatment of lung cancer;
2. It is necessary to evaluate the component of immunotherapy due to the accumulation of a polymeric complex in the spleen and determine whether this is a side effect;
3. To evaluate the possibility of reducing the course dose of 5-FU in the polymeric complex compared with an independent preparation by maintaining the concentration on the plateau.

One way or another, the transition from currently used low-molecular-weight antitumor drugs to polymer systems makes it possible for their combined use in one polymer carrier, which in practical medicine will lead to the optimization of antitumor therapy, increasing the level of specificity of the interaction of antitumor drug with tumor cells, reducing side effects and thereby expanding the therapeutic effect.

## 4. Materials and Methods

### 4.1. Method of Obtaining Poly(methacrylic acid) (PMAA)

Methacrylic acid (MAA) (Aldrich, St. Louis, MO, USA) was used to synthesize polymers. 2-phenylpropane-2-ilbenzodithioate (0.04 mol/L) was used for the synthesis of poly(methacrylic acid). Polymerization was carried out in a dimethylformamide solvent. Azobisisobutyronitrile (AIBN) (0.002 mol/L) was used as the initiator. The process was carried out at T = 70 °C in sealed ampoules, previously degassed. The resulting polymer was purified by re-precipitation with diethyl ether from a solution in methanol. The sample was then dried in vacuum until a constant mass was reached. A PMAA preparation with a narrow-variance distribution was thus obtained (Mn•10^−3^ = 31.8; Mw•10^−3^ = 37.3; Ð = 1.17).

### 4.2. Molecular-Mass Characteristics of the Polymer

The molecular-mass characteristics of the resulting compound were determined by gel permeation chromatography (PGC) in tetrahydrofuran at 40 °C. PGC was carried out on a Shimadzu Prominence LC–20VP liquid chromatograph with Tosoh Bio-science columns packed with polystyrene gel (mesh sizes 1 × 10^5^ and 1 × 10^4^ Å). Chromatograms were processed using LCsolution software (Version 1.25). Calibration was performed using narrow-variance PMAA standards. A differential refractometer was used as a detector. The removal of the end group of the agent was carried out as follows. Samples of poly(methacrylic acid) were dissolved in tetrahydrofuran, and then heated with a 10-fold excess relative to the number of groups of the agent, the radical initiator of benzoyl peroxide in sealed ampoules at a temperature of T = 90 °C. The removal of the agent group was confirmed by ^1^H NMR spectroscopy.

### 4.3. Laboratory Animals

In vivo experiments were carried out in outbred Wistar rats (body weight 180–200 g), which were grown in a breeding facility with free access to food and water at a natural light/dark cycle. The rats were quarantined for 14 days prior to experiments. 

The rats were kept in a certified breeding facility of the Central Research Laboratory of the Privolzhsky Research Medical University in compliance with Sanitary Norms and Regulations SP 2.2.1.3218-14. This study was approved by the local Ethic Committee of the Privolzhsky Research Medical University (Minutes no. 06 dated 14 April 2023). All experiments were carried out in compliance with the Guide for the Care and Use of Laboratory Animals (National Academy Press, Washington, DC, USA, 2011).

### 4.4. In Vivo Tumor Graft Model

An experimental neoplasm model was obtained by grafting PC-1 cancer cells, which were obtained from the Blokhin Cancer Research Center. The tumor is mucinous carcinoma originating from the biliary epithelium of the rat liver; the original tumor was hepatic cholangioma. The transfer began with anesthesia of the donor rat, then the subcutaneous tumor was cut out and crushed in a sterile Hanks solution in a ratio of 50 mg per 0.5 mL, resulting in a suspension of tumor cells. The resulting suspension of cells was completely injected into the recipient rat subcutaneously in the area of the anterior abdominal wall closer to the hind leg. Grafting was performed under isoflurane anesthesia. The day of cancer cell grafting was considered to be day 0 of tumor development. Based on the characteristics of the growth of the PC-1 tumor, the tumor node begins to form on the 16th–19th day after the grafting and begins to grow actively on the 29th–31st day. On the 30th day after the transfer, the animals were divided into groups: a control group, a group with 5-FU and a group with a 5-FU polymeric complex.

### 4.5. Production of a 5-FU Polymeric Complex

Polymeric complexes were obtained using commercial 5-FU (Ftorouratsil-LENS, 50 mg/mL, 20 mL, 10 ampoules, solution for intravascular administration (Veropharm, the town of Volginsky, Russia), PMAA, acetonitrile (KhIMMED, Moscow, Russia), and purified water.

PMAA was dissolved in water and added to a 5-FU solution in acetonitrile. The resulting mixture was incubated in the dark on a magnetic stirrer for 48 h. The solvent was removed, and the powder mixture was dried in a dry air oven at 40 °C to a constant weight [28].

The degree of 5-FU incorporation in the polymeric complex was determined by UV spectroscopy. UV spectra were recorded using a Shimadzu UV 1800 direct-current spectrophotometer (working range 190–1100 nm, wavelength accuracy ± 0.1 nm; Shimadzu, Kyoto, Japan). Substances to be tested were dissolved in water.

### 4.6. Pharmacokinetic Studies

Rats were injected intravenously with free 5-FU (4 mg/kg) or a polymeric complex (an equivalent 5-FU dose) once daily for 3 days. Blood samples (0.3 mL) were drawn from the tail vein at the following time points: prior to the first administration (baseline); 10, 20, 60, 180, 360, and 1440 min after the first administration; 10 and 1440 min after the second administration; and 10, 20, 60, 180, and 360 min after the third administration. To isolate the serum, blood samples were centrifuged at 3000 rpm for 10 min; the serum was collected in Eppendorf tubes and stored at −20 °C until use.

### 4.7. Biodistribution

The rats were sacrificed via decapitation after drawing the last pharmacokinetic blood sample. To study the biodistribution, the brain, liver, lung, kidney, tumor, heart, and spleen were isolated and washed with chilled isotonic saline. Each tissue (1 g) was homogenized in 3 mL of a phosphate buffer (pH 7.4). The homogenate was centrifuged at 6000 rpm at 4 °C for 30 min. The supernatant was collected in Eppendorf tubes and stored at −20 °C until use.

### 4.8. 5-FU Measurements in the Rat Plasma and Organs

Measurements were performed using an LC-20 Prominence liquid chromatograph (Shimadzu, Kyoto, Japan) with a SPD-M20A UV-Vis photodiode array detector and LCsolution software (Version 1.25) for data collection and processing. 

Chromatographic column: C18 Supelco 5 µm, 250 × 4.6 mm.

Mobile phase: Trichloroacetic acid aqueous solution (pH 3.5)–methanol (95:5, *v*/*v*).

Flow rate: 1 mL/min.

Thermostat temperature: 40 °C. 

Detection wavelength: 265 nm. 

### 4.9. Serum Sample Preparation

A serum sample (0.10 mL) was combined with 0.10 mL of 30% sodium hydroxide (aqueous solution), and the mixture was incubated at room temperature for 1 h. The mixture was neutralized by adding 0.10 mL of 50% phosphoric acid and centrifuged at 3000 rpm for 10 min. The supernatant (0.2 mL) was combined with 0.5 mL of methanol and centrifuged at 3000 rpm for 10 min. 

The supernatant was filtered through a 0.22 µm nylon filter; the filtrate (0.20 µL) was injected in the chromatograph.

### 4.10. Tissue Sample Preparation for 5-FU Concentration Measurement by HPLC

A methanol extract (1 mL) of a homogenized rat organ was combined with 0.25 mL of 30% sodium hydroxide (aqueous solution) and incubated for 10 min. The mixture was neutralized by adding 0.25 mL of 50% phosphoric acid and centrifuged at 3000 rpm for 10 min. The supernatant was filtered through a 0.22 mcm nylon filter; the filtrate (0.20 mcL) was injected in the chromatograph.

## 5. Conclusions

Our results demonstrate that incorporation in a polymeric complex changes the pharmacokinetics and biodistribution profile of 5-FU. The polymeric complex was shown to accumulate to a higher level in the lung and spleen. Further studies are necessary in order to verify the efficacy of the 5-FU polymeric complex. 

## Figures and Tables

**Table 1:** Peaks in the IR spectra of PMAA, 5-FU, and the 5-FU polymeric complex.

IR Spectrum Peaks	PMAA	5-FU	5-FU Polymeric Complex
1702 cm^−1^	-C=O carbonyl group	-	-C=O carbonyl group
1249 cm^−1^	-	C-F	
814 cm^−1^	-	deformation vibrations of the C-H pyrimidine ring	

**Table 2:** Average values of the volume and weight of tumors of intact laboratory animals and laboratory animals injected with the 5-FU polymeric complex.

	Intact Control	5-FU Polymeric Complex
Average tumor volume (mm^3^)	3069	989.80
Average tumor weight (g)	4.87	2.67

## Data Availability

The data presented in this study are available on request from the corresponding author.
